# Chronic Opioid Therapy and Opioid Tolerance: A New Hypothesis

**DOI:** 10.1155/2013/407504

**Published:** 2013-01-14

**Authors:** Joel S. Goldberg

**Affiliations:** Anesthesiology Service, Durham Veterans Affairs Medical Center and Department of Anesthesiology, Duke University School of Medicine, 508 Fulton Street, Durham, NC 27705, USA

## Abstract

Opioids are efficacious and cost-effective analgesics, but tolerance limits their effectiveness. This paper does not present any new clinical or experimental data but demonstrates that there exist ascending sensory pathways that contain few opioid receptors. These pathways are located by brain PET scans and spinal cord autoradiography. These nonopioid ascending pathways include portions of the ventral spinal thalamic tract originating in Rexed layers VI–VIII, thalamocortical fibers that project to the primary somatosensory cortex (S1), and possibly a midline dorsal column visceral pathway. One hypothesis is that opioid tolerance and opioid-induced hyperalgesia may be caused by homeostatic upregulation during opioid exposure of nonopioid-dependent ascending pain pathways. Upregulation of sensory pathways is not a new concept and has been demonstrated in individuals impaired with deafness or blindness. A second hypothesis is that adjuvant nonopioid therapies may inhibit ascending nonopioid-dependent pathways and support the clinical observations that monotherapy with opioids usually fails. The uniqueness of opioid tolerance compared to tolerance associated with other central nervous system medications and lack of tolerance from excess hormone production is discussed. Experimental work that could prove or disprove the concepts as well as flaws in the concepts is discussed.

## 1. Introduction

Chronic pain is one of the greatest causes of human suffering. Chronic pain becomes intractable when standard therapies fail to control the pain [1, 2]. In many societies and regulated by laws, chronic opioid therapy is reserved for patients who suffer from intractable pain [3, 4]. Common examples of diseases that lead to intractable pain include arachnoiditis, brachial plexus avulsion, thalamic syndrome, and multiple surgical traumas. Patients who suffer from these conditions are frequently referred to pain management physicians who may consider invasive therapies such as neurosurgical deafferentation, peripheral, spinal cord, or deep brain stimulation, or high-dose opioid therapy [5]. This paper discusses possible mechanisms of two problems of chronic opioid therapy, namely, tolerance and opioid-induced hyperalgesia.

## 2. Pain Is an Excitatory Process, and Opioids Are Inhibitory [6, 7]

**Figure 1:**
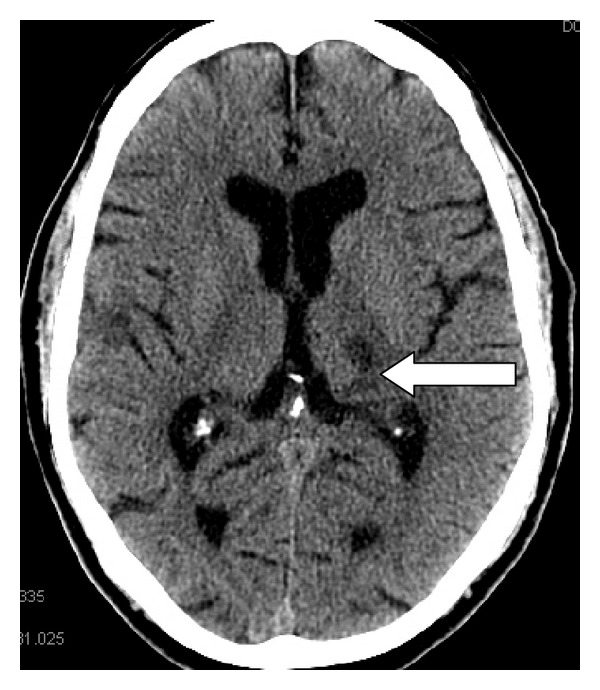
Hemorrhagic destruction of the thalamus is associated with intractable pain (white arrow).

**Figure 2:**
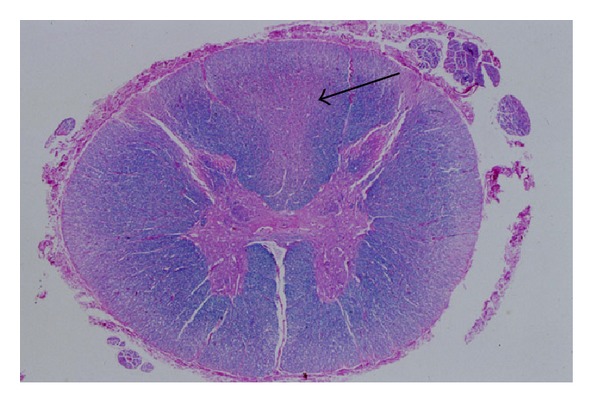
Destruction of posterior columns (arrow) from B 12 deficiency is associated with intractable pain. Subaute combined degeneration of *spinal cord*—B12 deficiency. pathology.mc.duke.edu. With permission.

From the first-order afferent receptor (mechanical, thermal, or chemical) through the ascending tracts to the thalamo, reticular, and mesencephalic relays to the final destination in the somatosensory cortices, anterior cingulated gyrus, and basal ganglia, pain is associated with a net excitatory stimulus. Numerous studies and clinical observations, including those from neural blockade, cordotomy, or rhizotomy, support this concept [8]. Destructive lesions of the third-order thalamocortical neurons that accompany thalamic syndrome or destructive lesions of the posterior columns of the spinal cord that occur in B 12 deficiency, tabes dorsalis, and multiple sclerosis produce pain because these neurons are inhibitory (Figures 1 and 2) [9]. Therefore, these destructive lesions produce a net excitatory response. Furthermore, stimulation of the neurons in the posterior columns can produce analgesia [10]. Numerous studies on the mechanism of action of opioids support clinical evidence that opioid effects have inhibitory influences on excitatory nociceptive pathways [11, 12].

### 2.1. Tolerance

#### 2.1.1. Neuroanatomic Correlates of Opioid Receptors and Ascending Nociceptive Pathways

Opioid tolerance can be defined as a decrease in analgesic response with increasing dose or frequency of administration. Tolerance is the greatest obstacle to the development of effective opioid treatment for intractable pain. Tolerance to endogenous opioids is often rapid, whereas tolerance to exogenous opioids is often delayed [13]. Opioids that are not associated with tolerance have not yet been developed. One hypothesis is that opioid tolerance is not only a function of ligand-receptor inefficiency but in addition may be caused by activation or upregulation (defined by an increased response to a stimulus) of non-opioid-dependent divisions of ascending pain pathways. This hypothesis is supported by the following observations.

**Figure 3:**
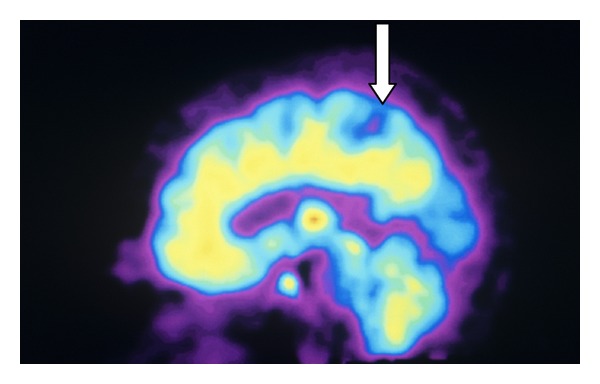
In vivo distribution of opioid receptors in human brain with decrease in receptors in area of S1 (arrow). Photo Researchers Picture Number: SF2688. Credit: Philippe Psaila/Photo Researchers, Inc. License: Rights Managed. Description: Opioid receptors. Colored sagittal Positron Emission Tomography. (PET) scan showing the normal distribution of opioid receptors in the human brain. By injecting a patient with an opioid tagged with carbon-11 (radioactive tracer), a color-coded scan is produced, showing the concentration of opioid receptors from red (highest) through yellow and green to blue (lowest).

**Figure 4:**
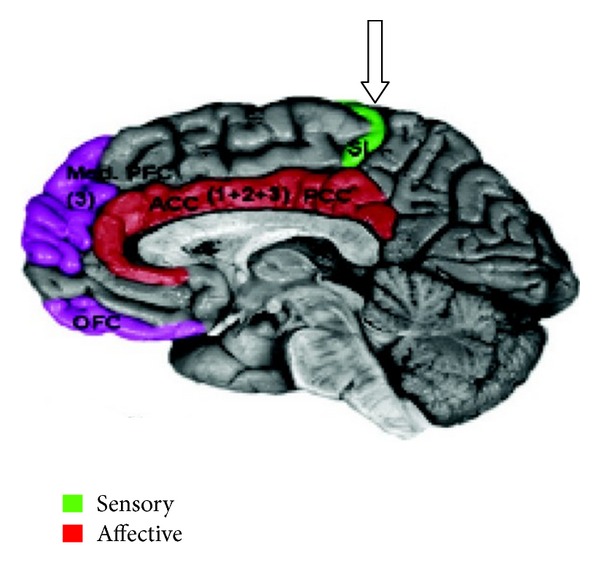
Somatosensory cortex (S1) of parietal lobe (arrow). Schematic of cortical areas involved with pain processing and fMRI cropped.jpg From Wikipedia, the free encyclopedia.

**Figure 5:**
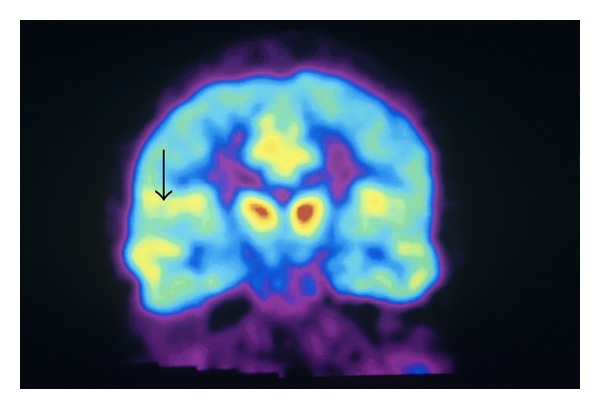
Opioid receptors in thalamus (red) and secondary somatosensory cortex, S2 (arrow). Photo Researchers Picture Number: SF2687. Credit: Philippe Psaila/Photo Researchers, Inc. License: Rights Managed. Description: Opioid receptors. Colored frontal Positron Emission Tomography. (PET) scan showing the normal distribution of opioid receptors in the human brain. By injecting a patient with an opioid tagged with carbon-11 (radioactive tracer), a color-coded scan is produced, showing the concentration of opioid receptors from red (highest) through yellow and green to blue (lowest).

**Figure 6:**
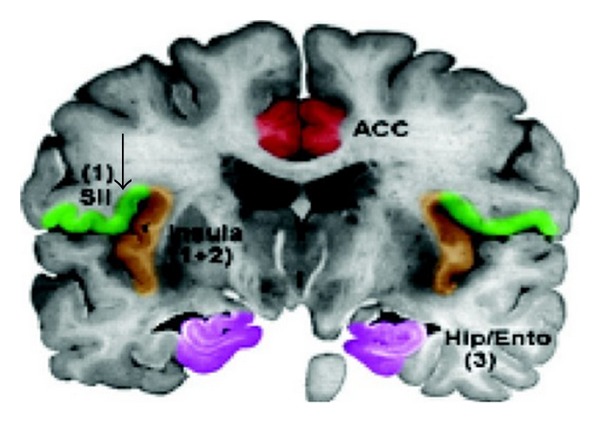
Secondary somatosensory cortex (S2) in green (arrow). Schematic of cortical areas involved with pain processing and fMRI cropped.jpg From Wikipedia, the free encyclopedia.

(1) *The Human Thalamocortical and Spinothalamic Tracts Have Divisions That Are Not Modulated by Opioids*. This observation has not been fully appreciated in the literature but is depicted in Figures 3, 4, 5, and 6.

Sensory fibers of the thalamocortical tract project to the primary somatosensory cortex (S1) and secondary sensory cortex (S2) [14, 15]. In humans, opioid receptors densely populate S2 but are very sparse in S1 [16]. In addition, in most studies, intrathecal or intravenous administration of opioids does not decrease the amplitude or latency of somatosensory-evoked potentials when monitored at S1 [17]. Therefore, a nociceptive division of the thalamocortical tract not modulated by opioids may be upregulated when opioids are administered. This could be viewed as a homeostatic mechanism that allows patients to maintain the ability to discriminate pain. Such upregulation of a sensory system is known to occur in the auditory perception of the blind and tactile and visual sensations of the deaf [18–22]. Deleterious effects associated with complete loss of pain sensation, whether genetic or acquired, as in Hansen's disease, are well recognized [23, 24]. If opioids completely abolished pain perception, patients would not be aware of traumatic events such as bone fractures, cholecystitis, and myocardial infarctions. Further support for the hypothesis that non-opioid ascending pain pathways are upregulated is found in the treatment of cancer patients who are medicated with very high doses of opioids. These patients experience profound analgesia after successful cordotomy with ablation of the lateral and medial spinothalamic tracts [8, 25].

**Figure 7:**
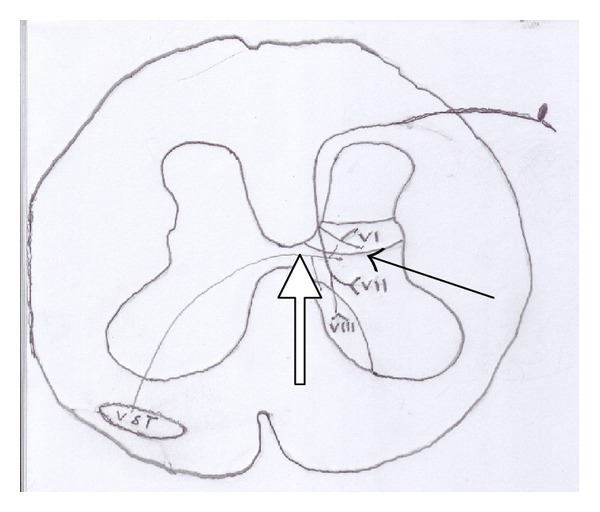
Ventral spinothalamic tract fibers (vst, white arrow) originate in non-opioid Rexed layers (VI–VIII) (dark arrow).

As shown in Figure 7, opioid receptors are rarely found in the origin of the ventral spinothalamic tract. Unlike the lateral and medial spinothalamic tracts, the ventral spinal thalamic tract does not project from Rexed layer II of the spinal cord where the highest concentrations of opioid receptors are found [26]. Instead, the fibers project from layers VI–VIII where very few, if any, opioid receptors exist, suggesting that this tract may be opioid independent and could upregulate [26].

(2) *Surgical Interruption of a Midline Dorsal Column Visceral Pain Pathway Produces Analgesia [27]*. Clinical reports confirm that a modified midline myelotomy can provide successful visceral pain relief especially in patients who have become tolerant to opioids. The analgesia produced by punctuate midline myelotomy that interrupts ascending fibers of the posterior columns has effectively controlled pain in patients suffering from pelvic pain secondary to malignancy [28]. It is not known whether this ascending nociceptive tract is regulated by opioids.

(3) *When Compared to Other G-Protein Ligands, a Unique form of Tolerance Develops after Opioid Administration*. Patients who suffer from hypersecretory states activated by G-protein receptors, such as hyperthyroidism, hyperparathyroidism, and pheochromocytoma, do not become tolerant to endogenous hormone ligands, and they need to be treated by lowering the production of these hormones. Although catecholamine tolerance has been reported in animal models, anecdotal reports from critical care physicians confirm that norepinephrine and dopamine administered as infusions rarely require major adjustments secondary to tolerance [29–31]. Opioids, either endogenous (endomorphin, enkephalin, endorphin) or exogenous (morphine, methadone, fentanyl), bind to G-protein receptors initiating a cascade that results in neuronal inhibition. Tolerance to opioid-induced constipation normally does not occur. Therefore, tolerance to endogenous G-protein receptor ligands may be specific to the central nervous system (CNS). Some have postulated that the milieu within the central nervous system is unique because of microglia effects, and that microglia activity contributes to opioid tolerance [32–34]. Tolerance develops to other G-protein ligands within the CNS, including cocaine, ethanol, benzodiazepines, amphetamines, and barbiturates, but the magnitude of the tolerance is usually less than that seen with opioids, and the perception associated with the tolerance is different because pain is not a consideration. 

### 2.2. Opioid-Induced Hyperalgesia [35–37]

Opioid-induced hyperalgesia is a condition that may occur after chronic administration of large doses of an opioid. It is characterized by a lowering of the pain threshold with an exaggerated response to painful and nonpainful stimuli [36]. One hypothesis to explain opioid-induced hyperalgesia is upregulation of the non-opioid-dependent divisions of the thalamocortical and/or spinothalamic tract. In contrast, neural blockade is rarely associated with tolerance or hyperalgesia because all proximal ascending tracts are inhibited.

### 2.3. Proof of Concept

The hypothesis that upregulation of non-opioid-dependent ascending sensory systems is a possible cause of opioid tolerance and opioid-induced hyperalgesia is novel, but recent reports have implied that this could be true [38]. This hypothesis could be proven by serial electrical recordings and imaging of the somatosensory cortex in patients treated with escalating doses of opioids to levels that produce signs of opioid tolerance. Considerable data are reported in the anesthesiology literature that describes somatosensory-evoked potential changes from various medications, and this information could be helpful [39, 40]. As opioid tolerance develops, functional MRI and PET scans may provide images that correspond to metabolic changes in the brain, and magnetic resonance spectroscopy could provide evidence for changes in glutamate and/or GABA concentrations in the somatosensory cortex [41–43]. Sensory stimulation during these studies may provide insight into the mechanisms of opioid-induced hyperalgesia. 

### 2.4. Implication for Therapy

Most pain practitioners acknowledge that opioid therapy in sufficient doses can ameliorate acute and chronic intractable pain, and addition of adjuvant medications such as antidepressants, anticonvulsants, and anti-inflammatory medication improves response. However, opioids alone cannot provide long-term relief because of tolerance, opioid-induced hyperalgesia, or side effects. Pain practitioners are also well aware that other nonopioid therapies, such as intravenous infusion of local anesthetics or ketamine, inhalation of nitrous oxide, or conduction block, provide reliable analgesia for patients suffering from intractable pain probably via a different mechanism than activation of opioid receptors. 

If upregulation of non-opioid-dependent ascending nociceptive pathways is shown to be a cause of pain from opioid tolerance or opioid-induced hyperalgesia, providers may consider adding GABA agonist to the medication regime of opioid-tolerant patients. Interestingly, our patients who suffer from intractable pain have learned that alcohol and benzodiazepines, both indirect GABA agonists, provide additional, but unsafe, analgesia when combined with opioids. In addition to increasing GABA, decreasing glutamate or blocking glutamate receptors could possibly attenuate the excitatory activity of non-opioid-dependent pathways. 

### 2.5. Possible Flaws in the Hypotheses

As espoused by most investigators, opioid tolerance is believed to be caused by ligand-receptor inefficiency [44]. Evidence suggests that endocytosis, downregulation of receptors, upregulation of P-glycoprotein, and mu/delta heterodimer formation may be causes of tolerance [45–47]. The hypotheses presented in this paper, regardless of how elegant they may appear, could be inaccurate. However, this author is not aware that in the current medical literature, location of human opioid receptors has been correlated with known ascending nociceptive pathways, and the significance of this observation extrapolated to possible etiologies of opioid tolerance and opioid-induced hyperalgesia.

## 3. Conclusion

Intractable pain is one of the leading causes of worldwide suffering. Opioids are the most consistent, efficacious, and cost-effective analgesics available. Tolerance to opioids limits their effectiveness, and some aspects of this phenomenon are unique to opioids when compared to other G-protein receptor ligand systems. One hypothesis of this paper is that some aspect of opioid tolerance is caused by homeostatic upregulation of non-opioid-mediated ascending nociceptive pathways, including the thalamocortical and ventral spinothalamic tracts and possibly the midline dorsal column tract. Another hypothesis is that upregulation of these tracts may contribute to opioid-induced hyperalgesia. As one observes in clinical practice, therapy with adjuvant agents combined with opioids, rather than opioids alone, produced the best outcome for the treatment of intractable pain. Aside from presumed stimulation of descending inhibitory tracts from some adjuvants, one possible explanation for these outcomes is that these therapies inhibit nonopioid ascending dependent tracts as well as opioid-dependent tracts. This paper offers a hypothetical explanation based on neuroanatomic correlation as to why adjuvants plus opioid therapy are most successful and single-opioid therapy usually fails. When shown a comparison of opioid and non-opioid ascending nociceptive dependent tracts, physicians and patients may develop a better understanding of their therapy.
